# Balanced Xylan Acetylation is the Key Regulator of Plant Growth and Development, and Cell Wall Structure and for Industrial Utilization

**DOI:** 10.3390/ijms21217875

**Published:** 2020-10-23

**Authors:** Mirza Faisal Qaseem, Ai-Min Wu

**Affiliations:** 1 State Key Laboratory for Conservation and Utilization of Subtropical Agro-Bioresources, South China Agricultural University, Guangzhou 510642, China; faisal.ali522@gmail.com; 2 Guangdong Key Laboratory for Innovative Development and Utilization of Forest Plant Germplasm, College of Forestry and Landscape Architectures, South China Agricultural University, Guangzhou 510642, China; 3 Guangdong Laboratory of Lingnan Modern Agriculture, Guangzhou 510642, China

**Keywords:** xylan, cell wall, acetylation, deacetylation, biosynthesis, esterases

## Abstract

Xylan is the most abundant hemicellulose, constitutes about 25–35% of the dry biomass of woody and lignified tissues, and occurs up to 50% in some cereal grains. The accurate degree and position of xylan acetylation is necessary for xylan function and for plant growth and development. The post synthetic acetylation of cell wall xylan, mainly regulated by Reduced Wall Acetylation (*RWA*), Trichome Birefringence-Like (*TBL*), and Altered Xyloglucan 9 (*AXY9*) genes, is essential for effective bonding of xylan with cellulose. Recent studies have proven that not only xylan acetylation but also its deacetylation is vital for various plant functions. Thus, the present review focuses on the latest advances in understanding xylan acetylation and deacetylation and explores their effects on plant growth and development. Baseline knowledge about precise regulation of xylan acetylation and deacetylation is pivotal to developing plant biomass better suited for second-generation liquid biofuel production.

## 1. Introduction

Xylan is the most abundant type of hemicellulose that occurs abundantly in cell walls of land plants, where it accounts for more than 30% of the dry weight, while in primary walls, it accounts for about 20% and its composition depends on the origin [1]. There is a lot of diversity in xylan structures as it depends upon the source of its origin. Generally, xylan is a heteropolymer with a backbone made of a β-(1→4)-D-xylospyranose backbone bearing 4-O-methyl-α-D-glucopyranosyl acid and α-L-arabinosyl and other monosaccharide side chains [2]. Depending upon the side chain on the xylan backbone, they can be divided into three major classes: glucuronoxylan, glucuronoarabinoxylan, and arabinoxylan. Glucuronoxylans are abundant in secondary walls of dicots and some non-grass monocots [2,3], glucuronoarabinoxylans are abundant in grasses and gymnosperms except members from *Gnetophyta* [4,5], and arabinoxylans are abundant in cereal grains [6,7]. In dicots and some non-grass monocots, glucuronoxylan made up to 25% of total weight of secondary walls. The glucuronoarabinoxylan is present in gymnosperm softwood [1] and grass species [2].

**Figure 1:**
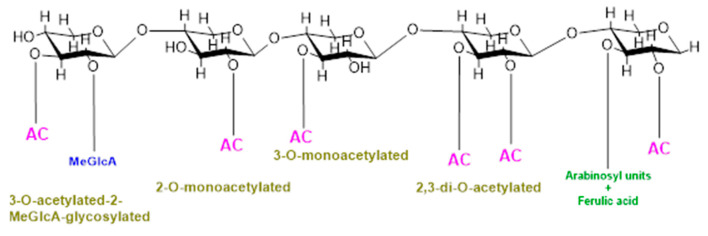
Types of xylan acetylation in woody plants and grasses.

In addition, backbone may also be substituted with O-linked methyl, acetyl, and feruloyl groups which protect polysaccharides from specific glycosyl hydrolases and cross-link cell-wall constituents controlling cell extensibility [8,9]. O-Acetylation is a common and prevalent method of xylan modification and is a ubiquitous substitution within hemicellulose families [10,11,12]. Cell wall polysaccharides are either mono- or di- acetylated as revealed by a study on ten out of fourteen cell walls constituting polysaccharides substituted with acetyl groups [10]. Furthermore, the positions of acetylation of these cell polysaccharides also vary, for example, xylose in xylan is acetylated at the O-2 and/or O-3 positions, galactose and/or mannose are acetylated at the O-6 position, and mannose in mannan/glucomannan is acetylated at the O-2 and/or O-3 positions [13,14]; fucose at O-6 and O-4 and galactose at the O-3 position in xyloglucan are also acetylated [15]. Acetylation of xylan can be of four different types, i.e., xylospyranose residues may be acetylated at the 2-O position, and thus, xylan will be called 2-O-monoacetylated; xylospyranose residues may be acetylated at the O-3 position and thus 3-O-monoacetylated; and xylospyranose residues may be acetylated at both the O-2 and O-3 positions and thus regarded as 2,3-di-O-acetylated. Finally, a xylospyranose residue may contain an acetyl group at the 3-O position and MeGlcA substitution at position O-3 is called 3-O-acetylated-2-MeGlcA-glycosylated xylan. In grasses, arabinose is attached at position three while the acetyl group is attached at position O-2 of xylopyronose residues [16] (Figure 1).

Although the exact degree of acetylation of cell-wall polymers is not yet known, many studies reveal that acetylation varies with plant type, tissue type, developmental stages, and cell wall [17,18,19,20,21,22]. For example, xylospyranose backbone of hardwood xylan is 70% acetylated at the C-2 and/or C-3 positions while softwood xylospyranose usually lack acetylation [11]. In poplar, as demonstrated by a recent study, the acetate can reach about 6.7% (w/w) of wood biomass [23]. Similarly, different plants or plant organs differ considerably in the types and content of xylan substitution, e.g., in the *Populus trichocarpa* stem, 63% of the total xylan is acetylated, of which 23.6% xylan possesses acetyl substitution at the O-2 position while 15.8% possesses acetyl substitution at the O-3 position, 14.8% xylan was substituted at both the O-2 and 3 positions, and 9.1% xylan has 3Ac-2GlcA substitution [24].

## 2. Difference in Substitution Patterns of Xylan

Glucuronoxylan has xylospyranose residues in its backbone connected via 1,4-linkages and contains acetyl and glucuronic acid or its derivatives as backbone substitutions and is common in many dicots [25]. In methylglucuronoxylan, the xylospyranose backbone is substituted with 4-O-methylgluconic acid at the O-2 position and has a common occurrence in birch and eucalyptus [19,26]. The specific positions of acetyl and methylgluconic acid on the xylan backbone have recently been demonstrated in study by [27]. Arabinoglucuronoxylan and glucuronoarabinoxylan are common in the arabinosyl and methylgluconic acid groups at the O-3 and O-2 positions, respectively. Both are different in the content of these two-sided chains along with the acetyl content. For example, non-acetylated arabinoglucuronoxylan from spruce has a higher O-4 methylgluconic acid substitution than arabinose [28,29]. The specific pattern of arabinose and methyl gluconic acid substitution in arabinoglucuronoxylan has recently been established [30]. Glucuronoarabinoxylan from sugarcane straw and bagasse xylans have either single or double substitution of arabinose with a lower methylgluconic acid content, while it is highly acetylated in hardwood and softwood [31]. The difference in the degree of xylan acetylation affects the physical and chemical properties of xylan, e.g., acetylation significantly affects the solubility as well as the water content of glucuronoxylans in aspen wood with small effect on molecular weight [32]. Furthermore, xylan acetylation enhances the thermal tolerance, mechanical strength, and hydrophobicity ideal for industrial utilization of xylan [32,33].

## 3. Substrate for Xylan Acetylation

Being the hub of all metabolic pathways, acetyl CoA regulates the metabolism of almost all essential nutrients and molecules needed to sustain life, including sugars, fats, and proteins [34,35,36,37,38]. It can be synthesized through multiple processes in the cell including glycolysis, the phosphoketolase pathway, and the Wood–Ljungdahl pathway in multiple cell organelles including plastid, mitochondrion, cytosol, and peroxisome [39,40,41,42,43]. Cytosolic acetyl CoA is a source of acetyl group for the acetylation of various types of metabolites, such as alkaloids, anthocyanins, isoprenoids, and phenols with a variety of commercial applications [44]. Although it was initially not clear which acetyl CoA pool is the exact source of xylan acetylation, recent studies have confirmed that cytosolic acetyl CoA is the sole donor of the xylan acetylation acetyl group [45]. A heteromeric enzyme ATP-citrate lyase (ACL) consisting of *ACLA* and *ACLB* subunits is responsible for synthesis of cytosolic acetyl-CoA as downregulation of antisense RNA of ACLA-1 in Arabidopsis leads to abnormal plant growth and reduced accumulation of multiple acetyl-CoA derivatives, e.g., stem cuticular wax and flavonoids in seeds [46]. Many In vitro studies have confirmed that polysaccharide esterases, i.e., XOATs, MOATs, and XGOATs associated with cell walls, can use acetyl-CoA as a substrate to transfer acetyl groups onto their respective oligosaccharide acceptors but that there was no evidence to support this argument in living plants until 2018. There are two problems with acetyl CoA acting as a donor of acetyl groups for xylan or other hemicellulose acetylation; firstly, there is no known acetyl-CoA-generating pathway in Golgi, and secondly, the lipid membrane is impermeable to acetyl-CoA [44]. Due to the impermeability of the Golgi membrane to acetyl CoA, there must be an intermediate that could facilitate the transport of acetyl CoA across the membranes. Experimental evidence for this confusion was provided from a study conducted by [45], that identified multi-transmembrane RWA proteins (*RWA1*, *RWA2*, *RWA3*, and *RWA4*) as facilitators for the transfer of acetyl CoA to the Golgi. In plants, *RWA* proteins mainly consist of two clades *AB* and *CD*, *RWAs* belonging to *CD* clade transport acetyl CoA to the Golgi for xylan acetylation in *Populus* [47]. The presence of multi-transmembrane proteins or domains in bacterial or plants polysaccharide O-acetylating systems involved in transmembrane transport of acetyl CoA and self-acetylating across membranes is not known yet [48].

## 4. Mechanism of Xylan Acetylation

**Figure 2:**
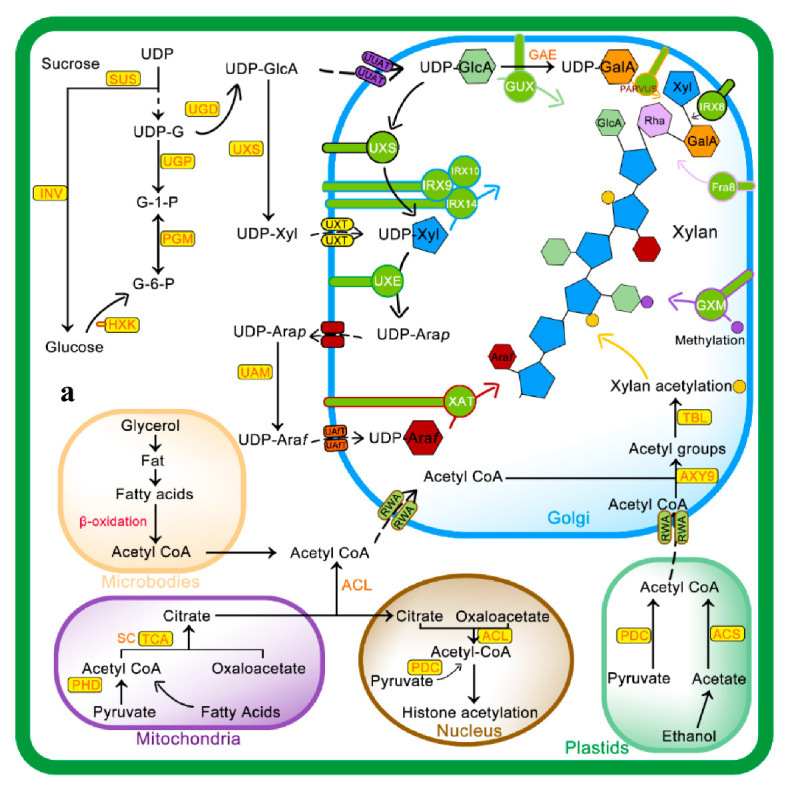

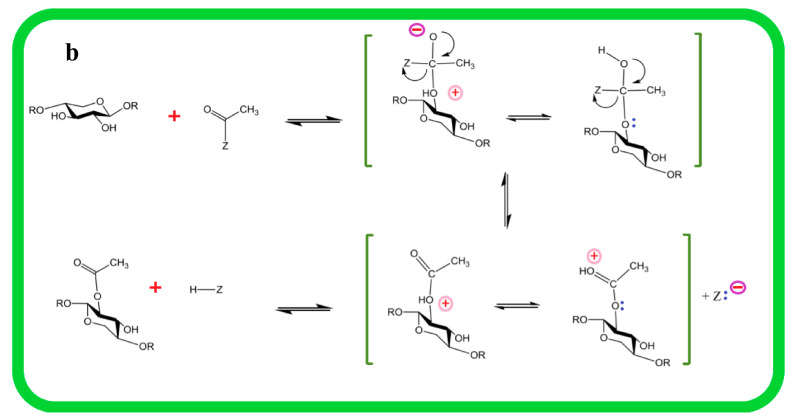
Mechanism of xylan biosynthesis and acetylation: (**a**) sucrose synthesized from photosynthesis is major source for UDP-glucose (UDP-G), which serves as a substrate for synthesis of various intermediates involved in xylan side chain or backbone synthesis. The Golgi is the actual site for xylan synthesis, so all substrates are transported to the Golgi via different membrane transporters. Acetyl CoA, a donor of acetyl group for xylan acetylation, is synthesized in different cell compartments i.e., microbodies, mitochondria, and plastids as well as in the cytosol from where it is transported to Golgi via Reduced Wall Acetylation (*RWA*) proteins and later incorporated to xylan. (**b**) Molecular mechanism of xylan acetylation adapted from [64,65]: attachment of acetyl to xylan involves nucleophilic attack of xylan OH group lone pair electrons on carbon atoms of the acetyl group to yield acetylated xylan. Abbreviations of all enzymes and intermediates are mentioned in Abbreviation section of manuscript.

Recent studies have shown that three main groups of proteins, i.e., *RWA* (Reduced Wall Acetylation), Trichome Birefringence-Like (*TBL*), and *AXY9* (Altered Xyloglucan 9), are involved in cell-wall polymer O-acetylation (Figure 2a,b). The polysaccharide O-acetyltransferases from the *TBL* family are well known for their ability to acetyl cell-wall polymers [49,50,51]. The enzymes encoded by the *TBL* family share the *TBL* and *DUF231* domains with the *Arabidopsis* Trichome Birefringence protein and contain the *Gly-Asp-Ser* and the *Asp-x-x-His* conserve motifs [10,52,53]. Till now, a number of members of the *TBL* gene family being studied for their role in regiospecific acetylation of xylan backbone, e.g., from Arabidopsis *TBL35* (*XOAT9*), *TBL34* (*XOAT8*), *TBL31* (*XOAT5*), *TBL32* (*XOAT6*), *TBL33* (*XOAT7*), *TBL30* (*XOAT3*), *TBL28* (*XOAT2*), *TBL3* (*XOAT4*), and recently *TBL10* is identified [20,49,50,51,54]. The structure and mechanistic details of *XOAT1* is published recently; it not only catalyzes acetylation of xylosyl residue at O–2 position but also facilitates nonenzymatic transfer of acetyl group to the O-3 position. The mechanism of *XOAT1* mediated acetylation involves a double displacement bi-bi mechanism involving a *Ser*-*His*-*Asp* catalytic triad which results in formation of the acyl-enzyme intermediate and uses an Arginine (*Arg*) residue for oxyanion hole formation. An important factor during transitional state of mechanism catalyzed by the *Ser*-*His*-*Asp* triad is oxyanion hole formation that regulate extra negative charge on acetyl group oxygen [55]. The *Agr* (*Arg*-219) residue in the conserved *RNQxxS* motif of *TBL*-block II is present in the active site and stabilizes negative charge during tetrahedral reaction intermediate formation [55].

*RWA* family proteins are considered an important component of wall polysaccharide acetylation as they are involved in the transfer of acetyl CoA from the cytoplasm to Golgi. *RWAs* contain multiple transmembrane helices similar to the transmembrane regions of the *CAS1* fungal protein glucuronoxylomannan acetylation [18]. Four members of the *RWA* family, i.e., *RWA1*, *RWA2*, *RWA3*, and *RWA4* have been reported in Arabidopsis, and any mutation in these causes a significant decrease in wall acetylation [56,57]. The Role of *AXY9* intermediate acetyl donor substrate has recently been proposed, with *GDS* and DxxH patterns homologous to the *TBL* family [58] and weak acetyl esterase activity [24]. *AXY9* may therefore act as an acetyl donor for xylan acetylation from other sources, i.e., pseudo-substrates, 4-methylumbelliferyl acetate, and p-nitrophenyl acetate [24], other than acetyl CoA and may form an acyl-*AXY9* intermediate, which may either act as a protein-activated acetyl donor or as an intermediate step in the formation of an unknown acetyl donor, but further research needs to confirm this [56].

As shown in Figure 2, initially, *RWA* proteins facilitate transport of cytosolic or acetyl CoA synthesized in other subcellular organelles to the Golgi [57,59]. Recent evidence suggests that *RWA* protein acetylates the proposed intermediate, i.e., *AXY9* [58], and subsequently, *TBL29* transfers the acetyl group to the xylan backbone [60,61]. The degree of xylan acetylation is regulated by a Golgi-localized *BS1* (BRITTLE LEAF SHEATH1) protein as the *BS1* mutant lacks specific xylan acetylation patterns [62]. The search for *BS1* orthologues in plants is on its way to understand occurrence and mechanisms of xylan deacetylation in the Golgi [63].

## 5. Deacetylation

The acetyl group is associated with the number of cell-wall polymers as the side chain and mechanism of polysaccharide acetylation is conserved across different organisms and kingdoms [10]. Acetylation has many important functions and is involved in many biologically important processes, although recent research has shown that not only acetylation but also deacetylation of cell-wall polysaccharides is vital to normal plant functioning [62]. Acetylation in plants is highly regulated, moderate deacetylation of xylan in aspen enhances saccharification without affecting the plant, while excess acetylation also increases saccharification efficiency but compromises plant growth and disruption of secondary wall structures [47,62]. In rice mutants, deacetylation disrupted interactions between cellulose and xylan, altered cellulose microfibril orientation, and resulted in thinner cell walls with less cellulose [66].

## 6. Mechanism of Deacetylation

**Figure 3:**
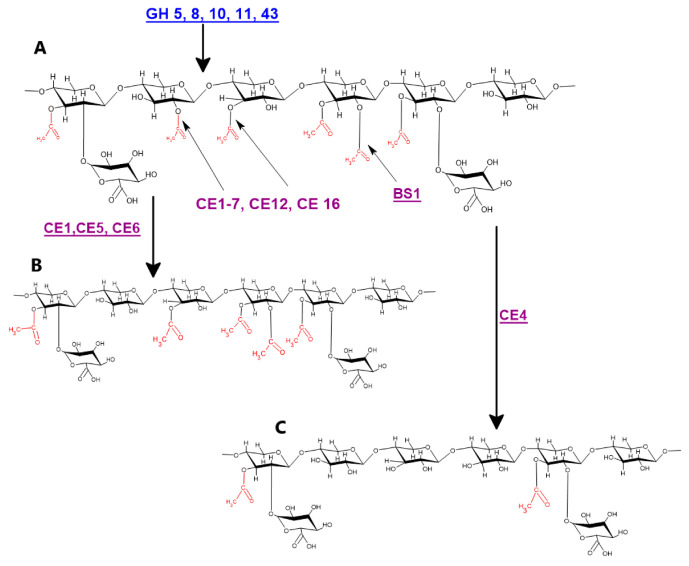
Genes and proteins involved in xylan deacetylation: (**A**) hardwood acetylated xylan; (**B**) CE1, CE5, and CE6 catalyzed deacetylation of hardwood xylan; and (**C**) CE4 catalyzed deacetylation of hardwood xylan. Blue colored genes indicate different endo-xylanases, while purple color indicates different xylan deacetylating enzymes.

Acetyl xylan esterases are the main enzymes involved in xylan deacetylation and are classified as carbohydrate esterases (CEs) and member of members of CE 1 to 7, and 16 have the ability to cleave the acetyl group from the xylan backbone (Figure 3) [11,67,68,69,70]. In addition, because of their sequence homology with acetyl xylan esterase, members of the CE12 protein family are also considered xylan acetyl esterases with some unknown functions. [71]. An acetyl xylan esterase belonging to the CE5 family isolated from *Hypocrea jecorina* when expressed in hybrid aspen under the control of the wood specific *PtGT43B* promoter caused 13% and 4% reduction in xylan acetylation and xylose, respectively, while glucose fraction was increased by 18% [72]. Carbohydrate esterase *FjoAcXE* isolated from *Flavobacterium johnsoniae* not only cleaves single or double acetylated xylopyranosyl residues but also efficiently cleaves internal 3-O-acetyl- xylopyranosyl linkages in (2-O-methylglucopyranosyluronic acid) 3-O-acetyl- xylopyranosyl residues. It also cleaves densely substituted and branched xylooligomers and significantly increases the activity of GH67 and GH115 α-glucuronidases [16]. Acetyl xylan esterases have been isolated from different sources including fungi, e.g., *Aspergillus*, *Myceliophthora*, *Neocallimastix*, *Penicillium*, *Trichoderma*, and *Volvariella*, and bacteria and plants, e.g., *Populus*, *Arabidopsis*, etc. have the ability to deacetylate many wall associated polysaccharides [73,74]. Acetyl xylan esterases (*AXEs*, EC 3.1.1.72) catalyze the hydrolysis of ester linkages between acetyl groups and xylan [75]. The *GDSL* lipase/esterase family consists of hydrolytic enzymes belonging to the *SGNH* hydrolase superfamily. More than 1100 members of the *GDSL* family are reported so far from twelve different fully sequenced plant genomes [76]. The specific role of *GDSL* lipases/esterases in xylan deacetylation was reported by [63,77]. A study of the rice (*Oryza sativa*) *brittle leaf sheath1* (*bs1*) mutant revealed that it has a defect in Golgi localization of the *GDSL* esterase and thus confirms the role of *GDSL* esterase in xylan acetylation. They also concluded that proper functioning of the *BS1* gene is necessary for proper xylan acetylation and secondary wall formation and patterning [63,78]. A rice brittle leaf sheath1 mutant defective in *GDSL* esterase was found to have the ability to deacetylate xylan backbone. Recently, DEACETYLASE ON THE ARABINOSYL SIDECHAIN OF XYLAN1 (*DARX1*) esterase was characterized and was involved in deacetylation of arabinose associated with arabinoxylan in grasses [62,77].

Furthermore, the association between xylan and cellulose is stabilized by many factors including the xylan backbone itself, the distance between substations on the backbone, and stabilizing effects of the adjacent GlcA [79,80,81]. The acetyl xylan esterases differ in their mode of action, and no member of acetyl esterases families with the ability to remove the acetyl group from the xylaopronosyl backbone acetylated at position three along with MeGlcA substation at position two of the same residue is known yet [67]. Thus, to overcome steric hinderance caused by MeGlcA and to break the complex association between wall polymers, a search for new xylan esterases with improved catalytic activity is the need of the hour [9]. This will serve as a reservoir of xylan deacetylases and will facilitate a better understanding of the xylan hydrolysis processes and conditions [71,82].

## 7. Importance of Xylan Acetylation and Deacetylation

### 7.1. Xylan and Cell-Wall Polymer Interaction

Xylan acetylation is a key player in the regulation of xylan interactions with cellulose and other wall polymers and also determines the hydrophobic nature of acetylated xylans, resulting in rigid wall conformation vital to normal plant functioning [23,83]. For example, xylan adsorption on the surface of cellulose decreases in the presence of high acetyl on the xylan backbone, thereby modulating the degree of alteration and pattern of xylan acetylation, lignin–xylan, or cellulose–xylan interactions [84,85].

Lignin–xylan association is important for biomass conversion, and recent studies on Populus have shown that this interaction is modulated by the degree of xylan acetylation. The optimization of xylan acetylation patterns is vital for optimizing pretreatments and enhancing biomass conversion [23]. Likewise, there are many reports that indicate inhibition of the hydrolytic activity of enzymes by excess acetate that further reduces the enzyme fermentation process and their accessibility to target polysaccharide molecules [67,86]. For example, acetyl groups completely and partially inhibit the activity of endoxylanase enzymes and also reduce acid hydrolysis [87,88,89]. The mono- or di-acetylation of xylopyranosyl residues in woody biomass alters hydrophobicity and causes steric hindrance, thus inhibiting or reducing the effective binding of hydrolytic enzymes to target polysaccharide [80,83,90,91]. Thus, the degree of xylan acetylation and patterning is vital for the determination of wall architecture and mechanical strength. Experimental evidences have shown that the degree of xylan hydrophobicity is affected by xylan acetylation, for example, deacetylated xylan absorbs more water due to extensive hydrogen bonding with water [32]. Weakly acetylated xylan is completely soluble in water, while highly acetylated xylan is only soluble in nonpolar solvents [92,93,94]. In conclusion, xylan acetylation inhibits xylan degrading enzymes and affects xylan cellulose and/or xylan lignin interaction and xylan hydrophobicity. Reducing xylan acetylation would therefore reduce the acetyl content that could improve the catalytic activity of xylanases and open the cellulose surface to give more access to xylanases.

### 7.2. Cellulose–Xylan Complex

Understanding of cellulose and hemicellulose interactions is important as it may affect tensile properties, e.g., flexibility, and is important for the different mechanical properties of wood-based materials.Developing a deeper understanding of how cellulose and hemicellulose interaction and how modification in one could have an impact on their association is important for the design of tailored composites with optimum properties and other industrial applications [95,96]. Evidences have shown that xylan backbones can adopt two confirmations, i.e., minor domains with threefold screw and major domains with twofold screw similar to two screw fold structure of cellulose [79]. The major domain xylan, rather than clumping with each other, enters into grooves present on the hydrophilic face of microfibrils, attaches them through hydrogen bonding, and forms a layer on the outer face of cellulose [79,81]. Alternative residues of major domain modifications are equally replaced by a conformation in which these groups are exposed to cellulose [97]. It has now been demonstrated that O-2 substitution of the xylan backbone facilitates formation of the twofold screw structure while O-3 substitution stabilizes the xylan cellulose complex [81]. The pattern of substitution on the minor domain is uneven and is closely located, forming a threefold screw conformation; the precise function of substitutions on minor domain is not known yet, but it is proposed that they are involved in establishment of hydrophobic pockets in the spaces between microfibrils [23,83,97,98].

**Figure 4:**
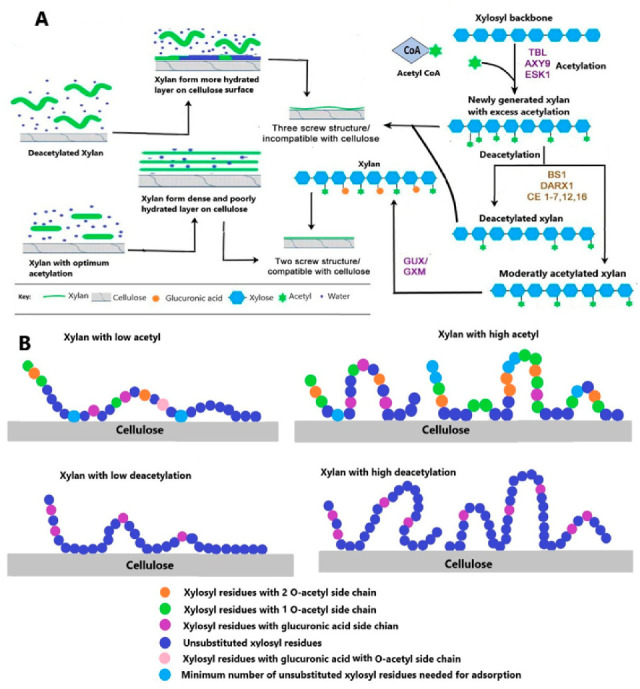
(**A**) Hypothesis of xylan acetylation and deacetylation and its binding with cellulose: the action of various Trichome Birefringence-Like genes (*TBLs)* in other acetylation catalyzing genes followed by GUX/GXM add acetyl and glucuronosyl (U) or 4-O-methylglucuronosyl (U^Me^) residues respectively to generate even-patterned xylan that is compatible with binding to the cellulose hydrophilic surface. In some cases, TBLs place an overloaded acetyl group on the same xylosyl residue, which is already substituted by a U^Me^, thus generating doubly acetylated xylosyl. Access of acetylation is removed by xylan esterase belonging to different classes and a member of GDSL esterases. The other half of the figure explains the effect of acetylation on a xylan cellulose complex. The xylan with excess acetylation or that is highly deacetylated forms a viscous and highly hydrated layer on the cellulose surface, while xylan with optimum acetylation forms a dense layer with tight bonding between xylan and cellulose. (**B**) The proposed mechanism of xylan adsorption extracted from *Eucalyptus* wood on bacterial cellulose surface modified from [100].

The importance of xylan acetylation and deacetylation in the regulation of cellulose and xylan interaction is established recently, as it is believed to regulate the hydration pattern of the xylan–cellulose complex vital for strength and rheological properties of cell wall [80,99]. Less branched xylan was best adsorbed on the surface of cellulose than highly substituted xylans [99,100]. Furthermore, the arabinosyl and O-acetyl substituted xylan have considerably low adsorption on bacterial cellulose surface [100]. Effective bonding of xylan with both hydrophilic and hydrophobic faces of cellulose microfibril is crucial for twofold helical screw conformation of xylan backbone. Alternative substitution of xylan back and twofold helical screw conformation are two important requirements for effective docking of xylan on hydrophilic faces of cellulose microfibril [83]. The role of acetylation on xylan cellulose complex formation is discussed in the next section of this review. The acetylated and deacetylated xylans differ in their adsorption on cellulose surface and thus have different surface morphologies on cellulose microfibrils [101]. A recent study revealed that acetylated xylan makes a rigid, less hydrated layer on surface of cellulose with two xylan residues per helical turn while deacylated xylan forms more viscous and swollen layers on the surface of cellulose with three residues per turn [102]. An investigation of xylan adsorption on bacterial cellulosic surface revealed that its adsorption is affected by size and substitution. Linear and unsubstituted conformation of xylan has higher adsorption compared to xylan with acetyl or arabinose substitution [100]. Generally, less substituted xylan make xylan–xylan aggregates with low solubility and thus adsorb efficiently on cellulosic surface [100,103]. High acetyl content in Eucalyptus xylan prevented xylan self-association and reduced absorption on bacterial cellulose surface [100]. Thus, precise xylan acetylation is necessary for its binding with cellulose and other wall polymers, and any change in acetylation pattern will not only affect its interaction with these polymers but will also cause misfolding of the xylan backbone [80,104]. The proposed model for regulation of xylan acetylation and deacetylation and for an effect on xylan cellulose complex and adsorption of xylan on cellulose surface are shown in Figure 4A,B.

### 7.3. Plant Structures and Development

Multiple plant cell-wall polymers have a range of functions, from regulation of plant growth and development, and transduction of water and nutrients to tolerance for environmental stresses. These diverse functions are controlled by incorporating acetyl to the cell-wall polymer network [10]. In comparison to other modifications, the biological function of O-acetyl substituents is not understood yet, although some evidence indicates that the degree and pattern of acetylation is modified during different plant growth and development stages [104,105]. Studies of various acetyl mutants suggest that strong reduction in xylan acetylation results in dwarfism, reduced mechanical strength of the stem, collapsed vessels, and stunted plant growth [53,58,61,106]. In aspen, a moderate decrease in xylan acetylation did not affect plant growth and improved biomass saccharification [73], while excess acetylation resulted in many structural abnormalities and increased saccharification in rice [62]. Plant development and secondary wall patterning in rice are affected by excess acetylation [78]. In rice, *OsTBL1* and *OsTBL2* are involved in monoacetylation of xylan, and the *tbl1/tbl2* double mutant shows a 55% reduction in xylan acetylation and reduced growth with varying degrees of dwarfism [78]. Arabidopsis *tbl3*, *tbl31*, *tbl32*, *tbl33*, *tbl34*, and *tbl35* single mutants show a small reduction in acetylation and no visible phenotype, but several double mutants of these genes like *tbl3/tbl31, tbl32/tbl33*, and *tbl34*/*tbl35* show 7–20% reduction in xylan acetylation (Table 1) [20].

Recombinant *AXY9* proteins showed weak acetyl esterase activity toward several pseudo-substrates [108]. The *Arabidopsis axy9* mutant showed a 70% reduction in acetylation, affecting the structure of various cell-wall polymers including xylan and xyloglucan (Table 1). Later, it was confirmed that dwarfed organs, a dark-green leaf color, and an extremely collapsed xylem in the *axy9* mutant were due to 80% reduction in xylan *O*-acetylation in stem tissues [58]. A 40% reduction in xylan acetylation, leading to reduction in plant growth and collapse in xylem vessel phenotype, has been reported in single loss-of-function alleles of the *TBL29/ESK1* xylan acetyltransferase [61,107,109,110]. A reduction in rosette size, plant height, and dark-green leaves is seen in *tbl29* mutants, but phenotypes can be reversed by inducing expression of the *AtGUX1* glycosyltransferase in vascular tissue. The probable reason for phenotypic rescue is replacement of missing acetyl-substituents in the *tbl29* mutant with functionally equivalent glucuronic acid moieties [111,112]. A subsequent study proved that reduced acetylation due to a defect in *tbl29* is not directly responsible for the collapsed xylem vessel phenotype, and other developmental changes as a suppressor mutation (*kaktus*) were able to complement all *tbl29* induced changes [108]. A similar *tbl29/esk1* suppressor mutation called *kaktus* was able to revert dwarfism and collapsed xylem phenotype induced by the change in *tbl29* [113]. A recent study found that expression of the MAX4 gene involved in the synthesis of *Methylcarlactonoate* (MeCLA) was reduced by *tbl29/esk1* suppressor mutation. Blocking *Methylcarlactonoate* biosynthesis can reverse all developmental and stress-related abnormalities caused by *TBL29/ESK1* loss of function without affecting its direct effect, i.e., reduced wall o-acetylation. As a result, we can infer that the reduced O-acetyl substituent is directly responsible for observed changes in morphology and development of *tbl29/esk1* mutants. Alternatively, by triggering a strigolactone hormonal pathway as a countervailing mechanism, plants may perceive defects in the structure of wall polymers and/or wall architecture [114]. A recent study aimed at accessing the effects of altered acetylation either by changing the expression of *RWA* genes or by post synthesis removal of acetyl xylan esterases on field productivity of 18 hybrid aspen lines suggests a 10–20% reduction in wall acetylation. This reduction resulted in growth, and genome and epigenetic changes in plants; one most prominent change was high frequency of dwarfism in which 17% resulted from the proposed link between acetyl metabolism and chromatin function [115]. Furthermore, many growth and plant developmental processes are regulated by polysaccharide-degrading enzymes that remove side chains from the cell-wall polysaccharide backbone and therefore affect solubility and binding with other cell-wall polymers. Furthermore, they may also act as proof-readers, correcting incorrect or extra substitutions on the polysaccharide backbone. For instance, *BS1* in Golgi removes excess acetate and regulates proper acetylation of the xylan backbone [77].

### 7.4. Stress Tolerance

The widespread occurrence of acetylation of cell-wall xylan has functional importance, as acetylation promotes interaction among cell-wall polymers, thereby contributing to the rigidity of the cell wall and facilitating different physiological functions [104]. The stiffness and rigidity of the cell wall conferred by xylan acetylation is necessary for protection of the plant against environmental and biotic stresses [10,116]. Another potential function of polymer O-acetylation is to protect plants against invading microorganism and environmental stresses. There are several mutants which show tolerance to stress for example; high freezing resistance was seen in *esk1* (*tbl29*) mutants [109], while the *pmr5* (*tbl44*) mutant showed resistance to powdery mildew [117]. Sensitivity to aluminum stress was increased in the mutant *axy4* (*tbl27*) mutant [118], and the *Arabidopsis rwa2* mutant showed increased resistance to the necrotrophic fungus *B. cinerea* and *H. arabidopsidis* [57,73]. The role of xylan acetylation in plant tolerance to stress is further confirmed by enhanced tolerance to necrotrophic fungi in reduced xylan acetylation transgenic plants due to overexpression of a fungal xylan acetyl esterase [119]. Rice *ostbl1* and *tbl1tbl2* mutants displayed susceptibility to rice blight disease, indicating that this xylan modification is required for pathogen resistance [78]. Engineered Arabidopsis plants with reduced acetylation when exposed to severe drought stress have high survival rate due to low water loss and upregulation of drought-responsive genes in the ABA-independent pathway, resulting in more drought-tolerant than wild types [120]. Cell-wall acetylation has a complex association with plant biotic stress tolerance, with reduced acetylation favoring tolerance to certain pathogens (fungi and bacteria), while in other cases, increased xylan acetylation favors tolerance to pathogens. In Arabidopsis, low levels of deacetylation enhance tolerance to *Botrytis cinerea*, a fungal pathogen, but not to the bacterial pathogen *Pseudomonas syringae* [57,119]. A study on role acetylation in abiotic stress tolerance revealed that reduced xylan acetylation counterbalances the deficiencies in immune response caused by an impaired β subunit of the G protein. In wild Arabidopsis plants, pathogen-induced stress was perceived by pattern recognition receptors that activate the G protein complex which scavenge reactive oxygen species produced as a result of oxidative damage. Finally, phosphorylation of *MAPK* and activation of other stress-related genes result in activation of pathogen-associated immunity. In G protein, Arabidopsis mutants with decreased xylan acetylation due to mutation in *esk1* result in activation of cell-wall-mediated damage-associated molecular pattern-triggered immunity that balances agb1-2 defective pathogen-associated immune responses. These evidences suggest that alteration in xylan acetylation patterns is essential for counterbalancing drought and pathogen stress-induced impairment of plant metabolism [121]. Furthermore, a recent study reported the effects of wall-based ester release on heat stress tolerance in plants. The authors believed that cell-wall-derived acetate could provide an alternative carbon source and could thus reduce decarboxylation of many important cell polymers. In addition, these significantly enhance CO_2_ content in chloroplast, produce multiple C_2_ intermediates such as acetyl-CoA, and regulate functions of various biopolymers during heat stress [122].

### 7.5. Cell Wall Esters and Environmental Services

Evidence from recent studies indicates that plant esters, i.e., methanol and acetic acid in addition to their role in the regulation of plant growth and development, are also involved in the sensing and signaling of pathways involved in cell-wall modification in response to various environmental stresses [122]. Being the first line of defense, the cell wall modification is a common phenomenon during plant response to stress. Many studies indicated that wall polysaccharides are highly modified during stress and common modification include methylation and acetylation [106,123]. Release of acetate from the cell wall and their subsequent transport into the environment via transpiration pull results in their release as volatile organic compounds or feed into central carbon and energy metabolism. Stress-induced signaling initiated via cell-wall modification results in damage-associated molecular patterns, which in turn activate plant immune response, suggesting a role of cell-wall-derived acetate in signaling and immune responses [116]. Although there is no empirical evidence for this argument, interdisciplinary research including biochemistry and metabolism of cell walls combined with plant physiology and biosphere can help to explain the underlying complex mechanisms [122]. High-latitude forests are of particular interest in responding to rapid global warming by the expansion of broad-leaf deciduous trees and the corresponding decline in evergreen conifer trees [124]. Due to the difference in the phenological cycles of the leaf and the composition of the wall, the timing, distribution, and magnitude of biosphere fluxes of volatile organic compounds, CO_2_, and H_2_O in these evolving forests may vary considerably in the future [122].

### 7.6. Importance for Wall Integrity and Bioconversion of Biomass

The role of acetylation in maintaining cell-wall structure is confirmed by many studies, concluding that acetyl groups act as barrier against enzymatic degradation of pectin and xylan [74,89,93,119,125]. Xylan acetylation generates some steric forces which prevent accumulation of xylan substrates during their synthesis and transport to the cell wall [126]. Acetylation also affects the xylan chain stiffness and the flexural properties of wood [127,128]. Similarly, excessive acetylation affects wood processing, pulping, and bioconversion; decreases fiber swelling; and inhibits the growth of microorganisms required for fermentation [23,129,130]. Xylan acetylation affects the lignocellulosic biomass that can be used for biofuel production [12]. Release of acetyl groups from xylan or another cell-wall polymer changes the pH of the reaction mixture and thus decreases glucose fermentation to ethanol [87,131]. From an industrial perspective, acetyl groups in the plant cell wall and other polymers play a vital role in their viscosity and gelation properties and thus their use in the food industry [132,133]. Understanding polysaccharide deacetylation is important as polysaccharides with low acetylation levels are required for downstream processing in biorefineries due to improved extraction of cell-wall material and excess release of toxic acetate that may affect many microbes such as yeast [10,56,57]. Deacetylated xylans or xylans with a low level of acetylation make tighter associations with cellulose, thus making isolation of entire wall materials difficult, meaning less material will be released after enzymatic hydrolysis. Mutants with higher levels of xylan deacetylation had thinner walls and less cellulose than the wild type [61,107].

A recent study reported that various degrees of acetylation can improve thermal stability of xylan from different plant sources and opens new avenues for the utilization of acetylated xylan from different biomass resources for use as thermoplastics and packaging [134]. Another study reported that chemical acetylation significantly improves the thermal stability of wheat bran feruloylated arabinoxylan-based biofilms without improving the mechanical or barrier properties [135]. Furthermore, in situ valorization of industrial xylans mainly extracted form poplar resulted in modified xylan with high solubility in water shear-thinning behavior and lower viscosity compared with those of the referenced hemicelluloses. All these properties of acetylated xylan alterations broaden their application in multiple industries [136].

## 8. Hypo- vs. Hyper-Acetylation

**Figure 5:**
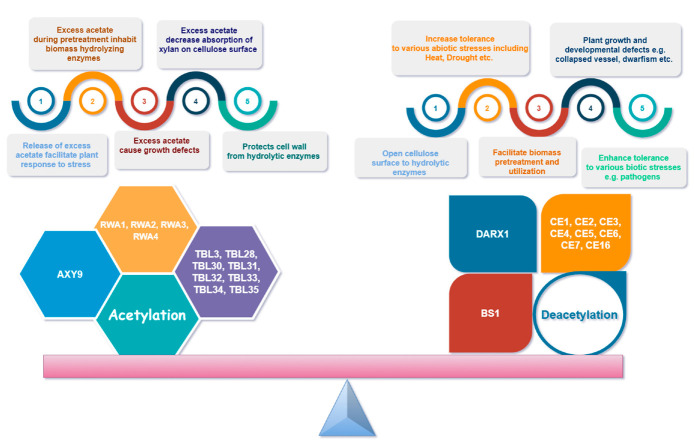
Importance of balanced xylan acetylation for plant development, environment tolerance, and polymer interactions.

Figure 5 demonstrates the importance of balance in xylan acetylation and deacetylation for plants and other factors. Xylan acetylation is of vital importance for plant growth, developmental processes, and stress tolerance and resistance [33,62,76,121,137]. An alteration in xylan backbone acetylation results in misfolding of the xylan backbone and thus affects cellulose–xylan complex formation [80,83]. Many studies suggest that the acetylation pattern promotes different organizations and hydrations of xylan cellulose complexes that can modulate the interaction strength and rheological properties of the cell-wall xylan–cellulose supramolecular complexes [30,100]. A very recent study showed that the layering pattern of xylan on cellulose is mainly affected by altered patterns of xylan acetylation and deacetylation. Thus, the presence of acetyl esters affects the supramolecular organization of xylan and its interaction with the surface of cellulose [102].

Both hypo- and hyper-acetylation of xylan has specific effects on plant growth and development as hyper-xylan acetylation in rice resulted in altered secondary cell wall patterning and abnormal development [62]. Early research on xylan acetylation also revealed that moderate (by approximately 20%) deacetylation either by inducing mutation [57,106] or by post synthetic deacetylation by an acetyl xylan esterase [138] is tolerated by herbaceous plants; however, strong deacetylation results in compromised growth as well as cell wall structure [127], as seen in case of *rwa1/2/3/4* and *tbl-29* mutants [61,106]. Moderating hypo-acetylation of xylan in hybrid aspen in addition to supporting plant survival and development also improved saccharification efficiency [139]. It is believed that xylan deacetylation after its synthesis (post synthetic) is a more effective approach than synthetic xylan deacetylation in the *Golgi*, which may result in excess glucuronidation [140] caused by the promiscuous activity of glucuronyl transferases *GUX1* and *GUX2* [80]. Thus, generally speaking, plant performance is proportional to the degree of deacetylation and the type of wall polymer modified may need to be optimized

Likewise, industrial use of xylan is also impacted by hypo- or hyper-acetylation. For example, moderate decrease in xylan acetylation by about 13–20% by *RWA* mutation or by acetyl esterases introduction can reduce biomass recalcitrance without compromising plant growth [47,139]. Interestingly, hyper-acetylation of *Populus* xylan also resulted in increased plant growth and stem volume coupled with reduced biomass recalcitrance [141]. Thus, alteration in the balance between xylan acetylation and deacetylation could impact secondary cell wall traits, biomass production, and recalcitrance [27,142]. Increased acetyl content is necessary for solid wood products, although the exact mechanism of increasing acetylation is not known yet. Overexpression of *TBL29* protein involved in xylan acetylation did not result in increase in acetyl content as demonstrated by study on *Arabidopsis* [61].

Thus, there are many open questions as far as xylan acetylation is required, for example, how to maintain the balance between xylan acetylation and deacetylation? Normally, deacetylation is believed to improve saccharification efficiency, but there is little evidence to suggest that increased acetylation may also improve saccharification efficiency. In order to understand the industrial applications of hemicellulose, a deeper insight into the detailed mechanisms of acetylation and deacetylation and its regulation is needed. It is also important to better understand the factors determining the degree and content of xylan acetylation in order to improve its bioconversion.

## 9. Conclusions

Xylan is an abundant hemicellulose and is a major component of grain, wood, and forage and therefore has a wide-ranging impact on human life. Although it is an abundant part of plant biomass, xylan is underutilized due to its enzyme resistance and structural complexity. Xylan acetylation plays a vital role in mediating noncovalent interactions between cell-wall polymers and in determining the nature and structure of the cell wall. Thus, the degree and pattern of xylan acetylation can affect the configuration and physiochemical properties of the cell wall and can provide the plant with mechanical strength and flexibility. Excessive xylan deacetylation promotes tight binding of xylan with cellulose, promotes self-association, and promotes plant defense against some pathogens. A reduced degree of acetylation is necessary for glycoside hydrolase-mediated hydrolysis of xylan, making it more accessible to degradation. The degree of acetylation is regulated by some acetyl esterases and deacetyl esterases. Understanding the control and accuracy of xylan acetylation and deacetylation is important for future improvements in plant biomass bioconversion. Although much research has been done on xylan acetylation and the enzymes responsible for xylan acetylation, the roles and functions of xylan esterases catalyzing deacetylation in higher plants is still lacking. Furthermore, additional research is required to uncover the exact mechanism of synthesis of the acetylation substrate (acetyl CoA), when and how enzymes are responsible for regulating O-acetylation and deacetylation of xylans, the mechanism and effects of the degree of acetylation and deacetylation of xylan on interaction with other polymers, and the mechanisms of sensing and response to environmental stresses.

## Figures and Tables

**Table 1:** Change in acetyl content and alteration in plant phenotypes in various plant mutants and transgenic lines.

Mutants	Percent Reduction in Acetyl Content	Change in Phenotype	References
*tbl29/esk1*	40	Reduction in rosette size, plant height and dark-green leaves	[78,107]
*esk1*	60	Dwarf plants with irregular xylem	[56,58]
*tbl32, tbl33 and esk1*	15	Severely collapsed vessels and stunted plant growth	[20]
*tbl1/tbl2*	55	Reduced growth	[78]
*axy9*	70	Change in xylan and xyloglucan structure	[58]
*axy9*	80	Dark-green leaf color and an extreme collapsed xylem	[58]
*rwa1/rwa3/rwa4 or rwa1/rwa2/rwa3*	20–30	Alteration in plant morphology	[58]
*rwa1/rwa 2/rwa 3/rwa 4*	42	Reduced acetyl coenzyme A transport	[58,106]
*rwa1/2/3/4*	60	Dwarf plants with irregular xylem	[56,58]

## Abbreviations

ACS	Acetyl-CoA synthetase
ACL	ATP-citrate lyase
ADP-Araf	UDP-L-arabinose Furanose form
AXY9	Altered Xyloglucan 9
FK	Fructokinase
FRA8	Fragile Fiber 8
Fru6P	Fructose 6-phosphate
GAE	UDP-GlcA 4-epimerase
GUX	Glucuronic Acid Substitution of Xylan
HXK	Hexokinase
INV	Invertase
IRX	Irregular Xylem
PDC	Pyruvate decarboxylase
PDH	Pyruvate dehydrogenase
PGI	Phosphoglucose isomerase
PGM	Phosphoglucomutase
RWA	Reduced Wall Acetylation
SC	Citrate synthetase
SUS	Sucrose synthase
TBL	Trichome Birefringence-Like
TCA	Tricarboxylic acid cycle
UAE	UDP-Ara 4-epimerase
UAM	UDP-Ara mutases
UDP-Api	UDP-D-apiose
UDP-Ara	UDP-L-arabinose
UDP-Arap.	UDP-L-arabinose Pyranose form
UDP-G	UDP-D-glucose
UDP-GalA	UDP-galacturonate
UDP-GlcA	UDP-D-glucuronate
UDP-Xyl	UDP-D-xylose
UGD	UDP-Glc dehydrogenase
UGE	UDP-glucose 4-epimerase
UXS and AXS	UDP-glucuronate decarboxylases
